# Bioinformatics Knowledge Map for Analysis of Beta-Catenin Function in Cancer

**DOI:** 10.1371/journal.pone.0141773

**Published:** 2015-10-28

**Authors:** İrem Çelen, Karen E. Ross, Cecilia N. Arighi, Cathy H. Wu

**Affiliations:** 1 Center for Bioinformatics and Computational Biology, University of Delaware, Newark, Delaware, United States of America; 2 Department of Biochemistry and Molecular & Cellular Biology, Georgetown University Medical Center, Washington, D. C., United States of America; University of the Sunshine Coast, AUSTRALIA

## Abstract

Given the wealth of bioinformatics resources and the growing complexity of biological information, it is valuable to integrate data from disparate sources to gain insight into the role of genes/proteins in health and disease. We have developed a bioinformatics framework that combines literature mining with information from biomedical ontologies and curated databases to create knowledge “maps” of genes/proteins of interest. We applied this approach to the study of beta-catenin, a cell adhesion molecule and transcriptional regulator implicated in cancer. The knowledge map includes post-translational modifications (PTMs), protein-protein interactions, disease-associated mutations, and transcription factors co-activated by beta-catenin and their targets and captures the major processes in which beta-catenin is known to participate. Using the map, we generated testable hypotheses about beta-catenin biology in normal and cancer cells. By focusing on proteins participating in multiple relation types, we identified proteins that may participate in feedback loops regulating beta-catenin transcriptional activity. By combining multiple network relations with PTM proteoform-specific functional information, we proposed a mechanism to explain the observation that the cyclin dependent kinase *CDK5* positively regulates beta-catenin co-activator activity. Finally, by overlaying cancer-associated mutation data with sequence features, we observed mutation patterns in several beta-catenin PTM sites and PTM enzyme binding sites that varied by tissue type, suggesting multiple mechanisms by which beta-catenin mutations can contribute to cancer. The approach described, which captures rich information for molecular species from genes and proteins to PTM proteoforms, is extensible to other proteins and their involvement in disease.

## Introduction

A wealth of knowledge relevant to the biological mechanisms of human disease, including information on protein-protein interactions (PPIs), protein post-translational modifications (PTMs), gene/protein expression and disease-associated mutations is contained in the scientific literature and bioinformatics databases. While it is challenging to collect information related to a gene/protein or disease of interest that is scattered across the scientific literature and housed in specialized databases with incompatible formats, development of systematic workflows to integrate and analyze information from disparate sources has the potential to uncover missing links and lead to new insights into disease etiology and treatment.

Combination of text mining tools to extract information from the scientific literature, curated databases, and ontologies, which enable structured representation of entities, relations, and concepts is a powerful strategy for knowledge integration. In previous work [1], we developed a bioinformatics framework for the construction of phosphorylation-centric networks that employed the Rule-based Literature Mining System for Protein Phosphorylation (RLIMS-P) text mining system to extract phosphorylation events from the scientific literature and information from phosphorylation and PPI databases (e.g., PhosphoSitePlus [2] and IntAct [3]), as well as the Protein Ontology (PRO) to represent phosphorylated protein forms (proteoforms; [4]) and the Gene Ontology (GO) [5] for functional annotation. Here, we extend that framework to additional information types and apply it to beta-catenin, a highly studied protein with a role in disease, in order to expand the applicability of the approach to disease-driver mechanisms.

Beta-catenin (gene name: *CTNNB1*) is a multi-functional protein that serves as both a cell adhesion molecule and transcriptional co-activator [6]. In metazoans, coordinated execution of these functions is critical for embryonic development and maintenance of tissue integrity in adult organisms. At the cell membrane, beta-catenin is a critical component of adherens junctions, structures that mediate cell-cell contacts in polarized epithelial tissues. In the nucleus, it acts as a transcription co-activator of T-Cell Factor/Lymphoid Enhancing Factor (TCF/LEF) family transcription factors, driving transcription of target genes. Free beta-catenin in the cytoplasm is rapidly targeted for ubiquitin-mediated degradation. The subcellular localization and stability of beta-catenin are regulated by extracellular cues as well as a complex network of PTM events. Signaling through the Wnt pathway, triggered by binding of Wnt ligand to cell surface receptors, stabilizes beta-catenin and promotes beta-catenin transcriptional activity. Conversely, phosphorylation of Ser-45 on beta-catenin by casein kinase I (CKI) followed by the sequential phosphorylation of Thr-41, Ser-37, and Ser-33 phosphorylation by the glycogen synthase kinase, *GSK3B*, creates a recognition site for the ubiquitin ligase *BTRC*, which ubiquitinates beta-catenin, targeting it for degradation by the proteasome. Dysregulation of beta-catenin activity is strongly correlated with cancer. Mutations that lead to beta-catenin disassociation from adherens junction and escape from ubiquitin-mediated degradation result in its translocation to the nucleus where it hyper-activates the transcription of its target genes, several of which have oncogenic activity [7].

In this report, we present a beta-catenin knowledge map constructed using our knowledge integration approach that includes molecular relations, protein sequence features, and proteoform specific functional information. By focusing on various sub-networks and features of the map, we addressed scientific questions regarding the biological function of beta-catenin and its role in cancer. Specifically, we characterized a group of beta-catenin interacting proteins whose expression is potentially controlled by beta-catenin; we proposed a mechanism for regulation of beta-catenin transcriptional activity by the cyclin dependent kinase *CDK5*, which was identified as a beta-catenin regulator in a large-scale miRNA-based knock-down screen of the kinome [8]; and finally, we examined beta-catenin cancer-associated mutations in conjunction with other sequence features to determine how beta-catenin activity may be altered in different cancer types.

## Results

### Construction and Characterization of the Beta-Catenin Knowledge Map

**Fig 1:**
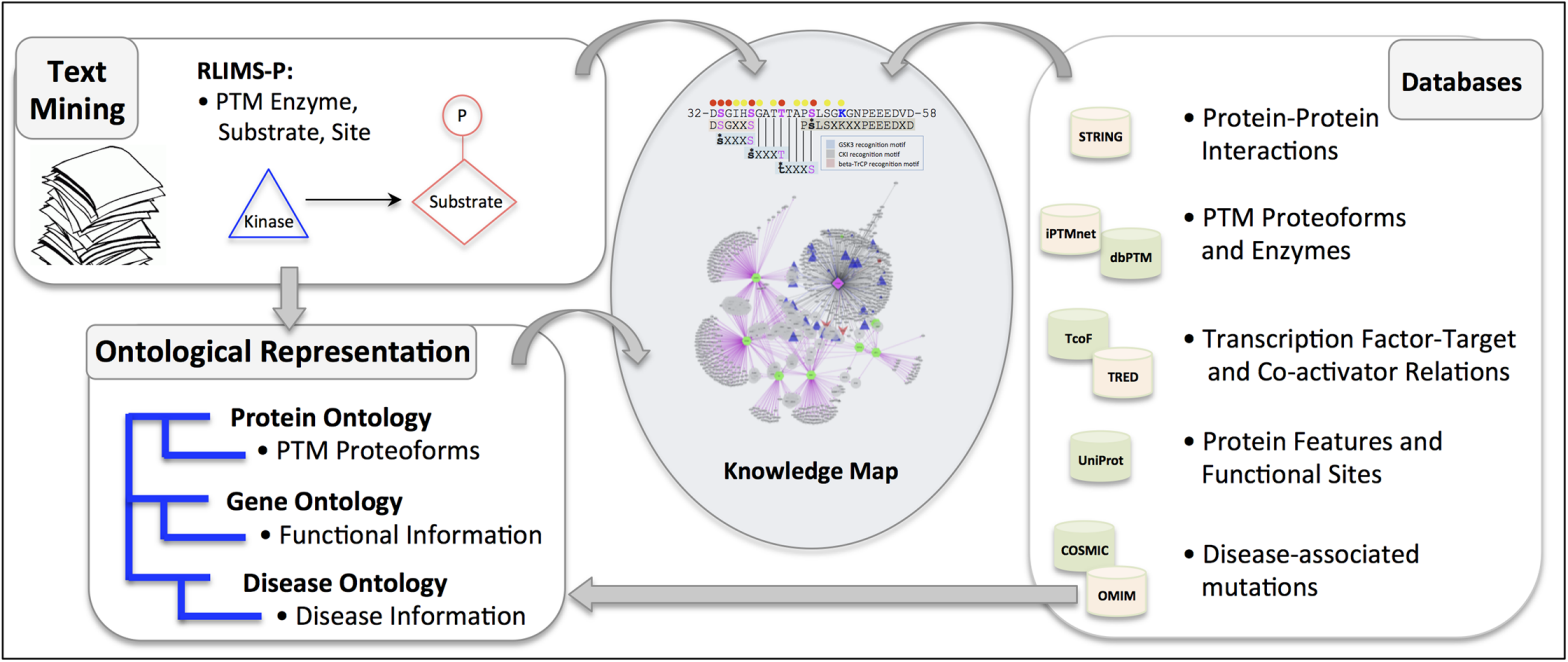
Bioinformatics framework for knowledge integration and construction of knowledge map.

Extending our previous work, we developed a bioinformatics framework to capture and integrate important molecular relations and attributes for proteins of interest for construction of knowledge maps (Fig 1). The overall approach involves text mining to detect molecular relations in scientific literature, integration of information from curated databases, and ontological representation of information. The literature mining tools and databases used can be tailored to the known roles of the protein under study; several examples of the resources that can be integrated are shown in Fig 1. Because PTMs are key regulators of beta-catenin activity, we emphasized incorporation of rich PTM information into the beta-catenin knowledge map. Phosphorylation events involving human beta-catenin were detected in the scientific literature with RLIMS-P, a literature mining system that identifies mentions of kinase, substrate, and phosphorylation site in text [9]. The beta-catenin PTM proteoforms described in the literature were represented in PRO [10] and annotated with functional information using GO terms [5]. In total, we identified 13 human beta-catenin proteoforms phosphorylated on various combinations of 15 different sites (eight serines, two threonines, and five tyrosines). Additional information on beta-catenin phosphorylation, acetylation, and ubiquitination, including PTM enzymes and sites, was obtained from bioinformatics databases. We only integrated information from manually curated databases with experimental validation (as opposed to results of prediction tools), along with clear links to supporting evidence, preferably to articles in the scientific literature. For phosphorylation information, we used our recently developed iPTMnet database (http://proteininformationresource.org/iPTMnet/), which provides a unified presentation of PTM information text-mined from the scientific literature and from multiple high-quality curated databases, including PhosphoSitePlus [2]—and Phospho.ELM [11]. To develop a comprehensive view of the role of beta-catenin in the regulation of gene expression and disease development, we expanded the knowledge map to include beta-catenin interacting proteins, including transcription factors co-activated by beta-catenin and their targets, as well as beta-catenin sequence features such as PTM enzyme binding sites and cancer associated mutations.

**Fig 2:**
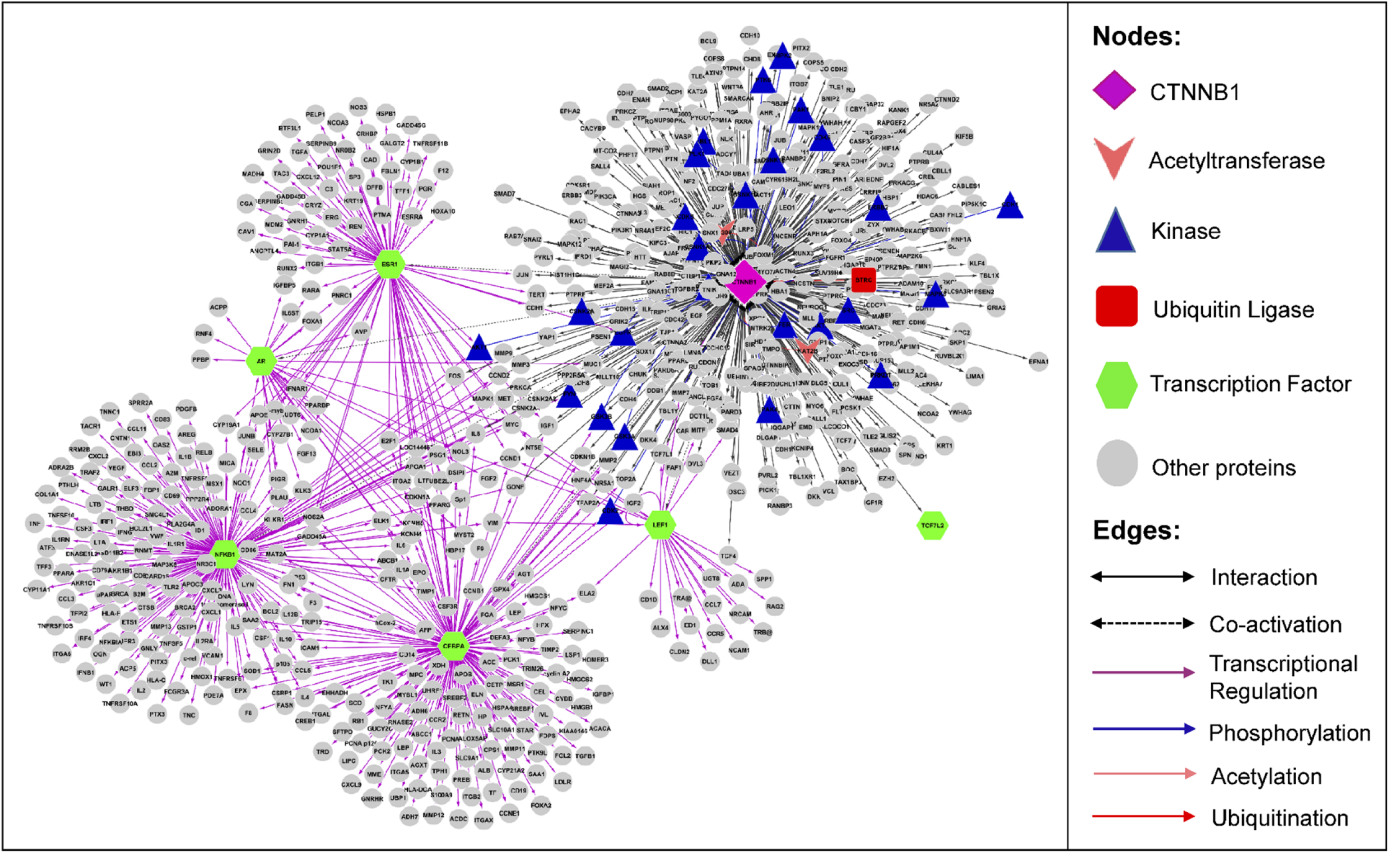
Beta-catenin biological network. The network depicts connections among beta-catenin (CTNNB1) PTM enzymes, interacting proteins, and transcription factors co-activated beta-catenin and their targets.

**Fig 3:**
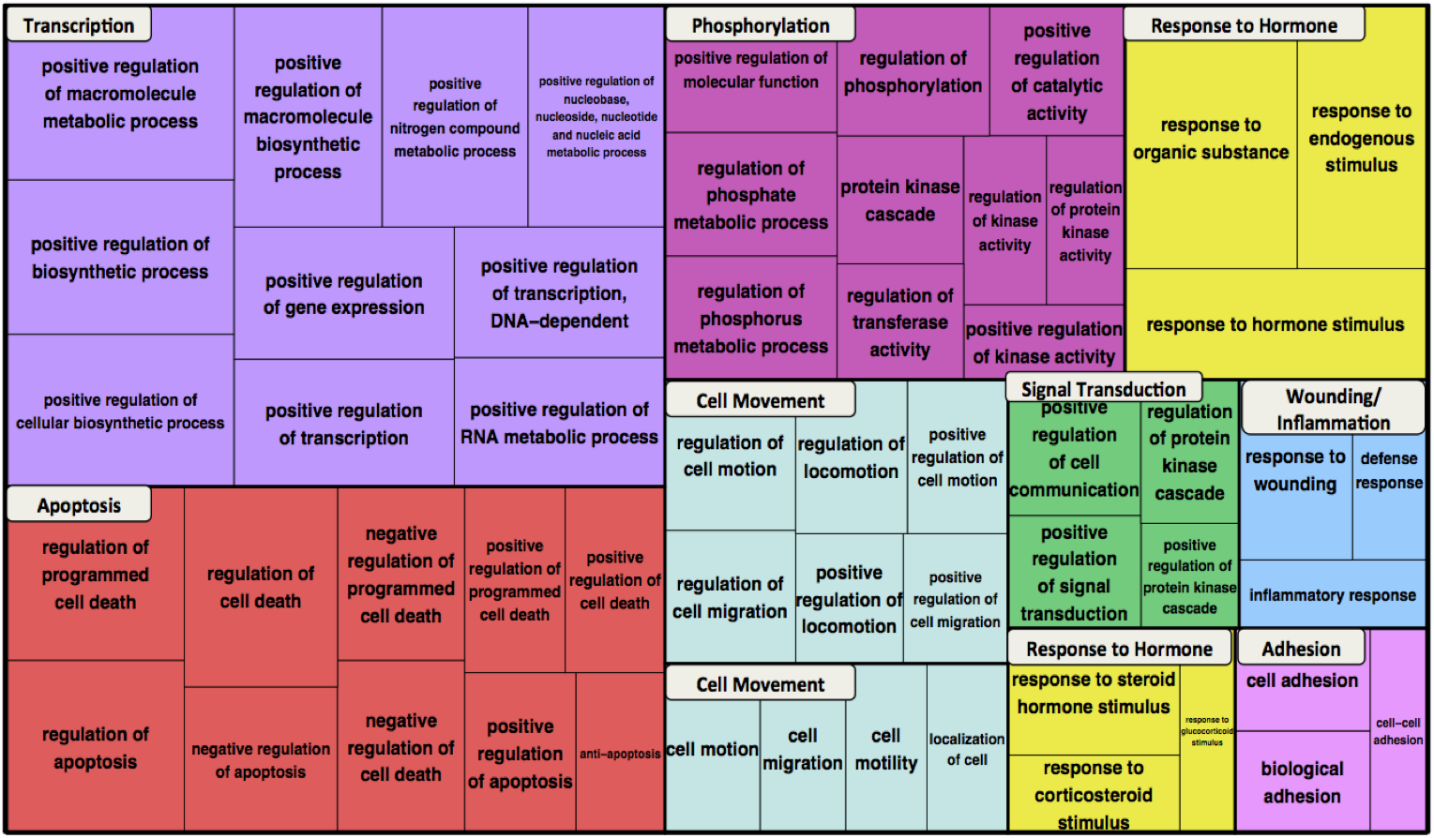
Functional enrichment of GO Biological Process terms for the beta-catenin network. Enriched terms were grouped into functional clusters. The most highly enriched terms for the top ten clusters are shown in the treemap, represented as different colored blocks. For each term, box size reflects the p-value of the term enrichment.

A network of beta-catenin molecular relations is shown in Fig 2. It consists of 727 distinct proteins participating in 861 relations (S1 Table), including six transcription factors co-activated by beta-catenin (dotted black edges), 445 transcription factor-target relations (purple edges), 381 beta-catenin PPIs, and 29 PTM enzyme-beta-catenin relations (26 phosphorylations (blue edges), two acetylations (pink edges), and one ubiquitination (red edge)). Integration of such knowledge for the protein of interest not only provides extensive information but also enables bridging the gaps in the knowledge about the protein at the systems level. For example, a researcher can identify the missing parts of a potential signaling pathway, transcription factor-target feedback mechanisms, or complex regulation of multi PTMs (see below). If the network comprehensively captures biologically relevant beta-catenin molecular relationships, then the biological processes associated with the network nodes should be reflective of the known roles of beta-catenin. To verify this, we performed GO term enrichment and functional clustering analysis, which groups enriched terms based on the assumption that terms associated with similar sets of genes are likely to be related to each other [12]. Highly enriched terms from the top ten clusters are shown in the treemap in Fig 3. The canonical biological processes in which beta-catenin is known to participate—transcription, cell movement, and cell adhesion—are represented in the top ten clusters. Clusters of phosphorylation and signal transduction terms likely reflect the focus of the network on beta-catenin PTM, particularly phosphorylation. The remaining clusters include response to hormone, wounding/inflammation, and apoptosis, which have all been associated with Wnt/beta-catenin signaling in the literature [13–15]. Thus, the relations in the network capture the most salient molecular relationships of beta-catenin.

### Identification of Potential Beta-Catenin Transcriptional Feedback Mechanisms

Proteins within the network that participate in more than one molecular relation are of special interest because they can provide insight into the regulation of beta-catenin and the coordination of its multiple functions. To demonstrate this idea, we used the beta-catenin network to identify proteins that might be involved in transcriptional feedback loops with beta-catenin. Feedback mechanisms, in which the product of an expressed gene stimulates (positive feedback) or suppresses (negative feedback) the transcriptional program that controls it, play a critical role in cellular transcriptional regulation. For example, the transcription factor *LEF1*, has been shown to participate in a positive transcriptional feedback loop with beta-catenin. In colon cancer cells, beta-catenin/*LEF1* complexes drive transcription of the full-length *LEF1* isoform that can bind beta-catenin at the expense of a dominant negative isoform. The expressed *LEF1* then associates with beta-catenin to drive further expression of full-length *LEF1* [16].

**Fig 4:**
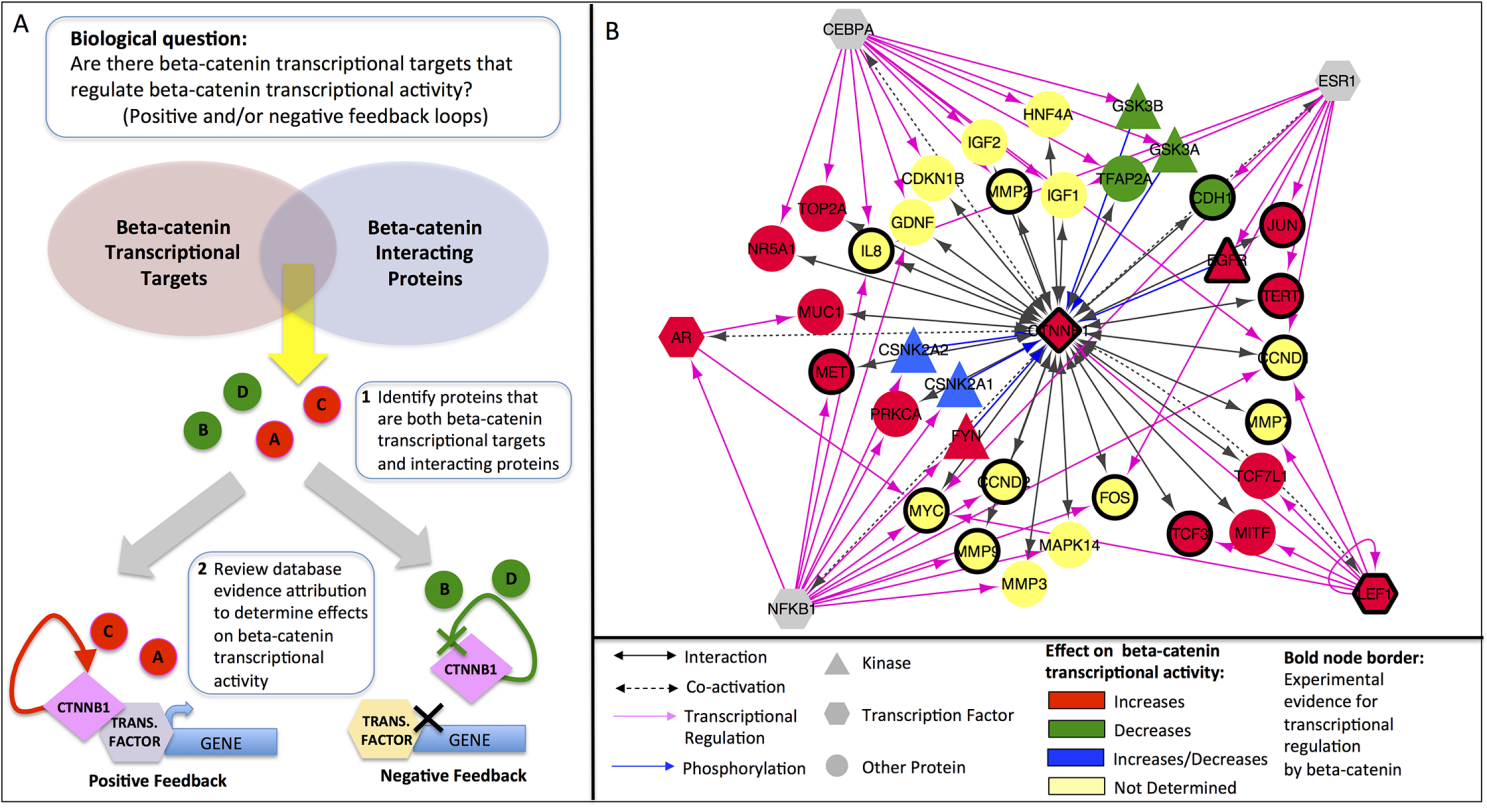
Beta-catenin sub-network for transcriptional feedback analysis. (A) Workflow for identifying beta-catenin transcriptional targets that affect beta-catenin transcriptional activity, thereby participating in positive and/or negative feedback loops. (B) Sub-network of beta-catenin interacting proteins whose expression is regulated by a transcription factor co-activated by beta-catenin (“target-interactors”). Node fill color indicates the effect of the interacting proteins on beta-catenin transcriptional activity. Nodes with heavy borders represent genes for which there is experimental evidence of transcriptional regulation by beta-catenin.

As outlined in Fig 4A, we reasoned that potential mediators of feedback mechanisms could be found among those proteins that are both beta-catenin transcriptional targets and beta-catenin interacting proteins. We identified a sub-network of 35 beta-catenin interacting proteins (including six beta-catenin kinases and two transcription factors co-regulated by beta-catenin) as well as beta-catenin itself that are targets of a beta-catenin regulated transcription factor (Fig 4B). Thus, these are proteins that could potentially be regulated at the expression level by beta-catenin and also modulate beta-catenin function. We will refer to these proteins as “target-interactors” to reflect this dual role.

Five different transcription factors regulate the expression of the target-interactors: *LEF1*, a member of the TCF/LEF family transcription factors; the androgen receptor *AR*; the CCAAT/enhancer binding protein C*EBPA*; the estrogen receptor *ESR1*; and the nuclear factor-kappa-B p105 subunit (*NFKB1*). With the exception of *LEF1*, these transcription factors are not completely dependent on beta-catenin for activity; they can work with other co-regulators or initiate their own transcription. Thus, we consulted the literature to determine what is known about the contribution of beta-catenin to the transcription of the target-interactors we identified. Fifteen of the target-interactors plus beta-catenin itself have either been shown to be regulated by beta-catenin in small-scale studies (the Wnt Homepage (http://web.stanford.edu/group/nusselab/cgi-bin/wnt/target_genes); Herbst et al., Table 1 [17] or were regulated by beta-catenin in at least two of three colon cancer cell lines examined in a recent genome-wide study [17]. This group includes the majority (6/8) of the *LEF1*-regulated target-interactors, and also multiple targets of *ESR1*, *NFKB1*, and *CEBPA* (Fig 4B, nodes with bold borders). Ten genes (*CCND1*, *JUN*, *CCND2*, *EGFR*, *LEF1*, *MET*, *MMP7*, *MYC*, *TCF3*, *TERT*, and beta-catenin itself (*CTNNB1*)) were up-regulated by beta-catenin and two (*FOS* and *CDH1*) were down-regulated; for the remaining genes, the directionality of the beta-catenin effect was not reported.

Not all proteins that interact with beta-catenin necessarily affect its transcriptional activity. Therefore, the next step toward identifying potential transcriptional feedback mediators was to determine what effect, if any, the target-interactors had on the transcription regulation function of beta-catenin (Fig 4A). We searched for information addressing the effect of the target-interactors on beta-catenin transcriptional activity using the text mining option of the STRING database [18] and by manually reviewing the literature cited by STRING as evidence for the interaction. For the target-interactors that are beta-catenin kinases, we also searched the functional annotation of the beta-catenin proteoforms in PRO that are phosphorylated by these kinases. Based on this information, we identified 14 target-interactors that increase (Fig 4B, red nodes) and four that decrease (Fig 4B, green nodes) the transcriptional regulatory activity of beta-catenin.

The target-interactors that potentially increase beta-catenin transcriptional regulatory activity include beta-catenin itself and several transcription factors co-regulated by beta-catenin: three TCF/LEF family members (*TCF3*, *TCF7L1*, and *LEF1*), *AR*, and *MITF*, which controls transcription of melanocyte-specific genes [19].

Two beta-catenin kinases—*FYN* and *EGFR*—also increase beta-catenin transcriptional activity. *FYN* phosphorylates beta catenin on Tyr-142 and *EGFR* phosphorylates beta-catenin on Tyr-654. Proteoform specific annotation in PRO indicates that Tyr-654-phosphorylated beta-catenin has enhanced transcription-related functions (PR:000044478). Moreover, Tyr-142 phosphorylation decreases beta-catenin association with the adherens junction, by inhibiting binding to alpha-catenin (PR:000036860), respectively. Reduction of association with the adherens junction increases the pool of beta-catenin available for transcriptional regulation in the nucleus. Similarly, two other target-interactors—*MET* and *MUC*—dissociate beta-catenin from the adherens junction and promote its translocation to the nucleus [20,21].

The target-interactors that decrease beta-catenin transcriptional regulatory activity act via several mechanisms: i) by promoting beta-catenin degradation (e.g., *GSK3B* phosphorylates beta-catenin on N-terminal sites (PR:000035772) that promote its association with the ubiquitin ligase *BTRC* and subsequent degradation); ii) via interaction with “inhibitors” in the nucleus (e.g., *TFAP2A* directly inhibits beta-catenin co-activator activity by forming a complex with beta-catenin and the adenomatous polyposis coli (*APC*) protein in the nucleus [22]); and iii) by increasing beta-catenin association with the adherens junction, thereby sequestering it away from the nucleus (e.g., E-cadherin (*CDH1*) associates with beta-catenin at the adherens junction [6]). It is important to note that beta-catenin transcriptional activity encompasses both its co-activator and co-repressor functions. Thus, given that beta-catenin has been shown to repress *CDH1* transcription, *CDH1* sequestration of beta-catenin away from the nucleus may actually result in an increase in *CDH1* expression.

Interestingly, casein kinase II (*CSNK2A1* and *CSNK2A2*, Fig 4B, blue nodes) appears to be capable of participating in positive or negative feedback depending on which sites on beta-catenin it phosphorylates. Phosphorylation of beta-catenin on Thr-393 increases its co-activator function (PR:000044474) whereas phosphorylation of beta-catenin on Ser-29, Thr-102, and Thr-112 (PR:000037187) leads to its association with the adherens junction and destabilization through increased association with the kinase *GSK3B*.

### Analysis of Kinase Signaling for Regulation of Beta-Catenin Transcriptional Activity

Multiple kinome-wide small interfering RNA (siRNA) knock-down screens have been performed to understand the impact of kinase signaling pathways on beta-catenin activity and sub-cellular distribution. One such study identified a group of kinases that seems to positively regulate beta-catenin co-activator activity under normal conditions [8]. One of the kinases identified, but not further characterized in the study, was *CDK5*. *CDK5* is a member of the cyclin-dependent kinase family of protein kinases implicated in the development of the nervous system and neuronal cell survival [23]. Recently, *CDK5* has been shown to participate in numerous biological processes outside of the nervous system, including some of the same processes—transcription, cell proliferation, and cell adhesion—that are regulated by beta-catenin [24]. CDK5, like beta-catenin, has also been implicated in tumorigenesis. For example, CDK5 promotes cell migration and invasion in pancreatic cancer cells, and inhibition of CDK5 suppresses pancreatic tumor growth and metastasis [25]. Activation of ERBB2 (Her2) and CDK5 and subsequent phosphorylation of the STAT3 transcriptional regulator is associated with cell proliferation in medullary thyroid tumors [26]. Finally, phosphorylation of androgen receptor by CDK5 plays a role in driving prostate cancer growth [27]. Thus, we were interested in using the beta-catenin knowledge network to identify possible links between *CDK5* and positive regulation of beta-catenin co-activator function that could be relevant to cancer.

**Fig 5:**
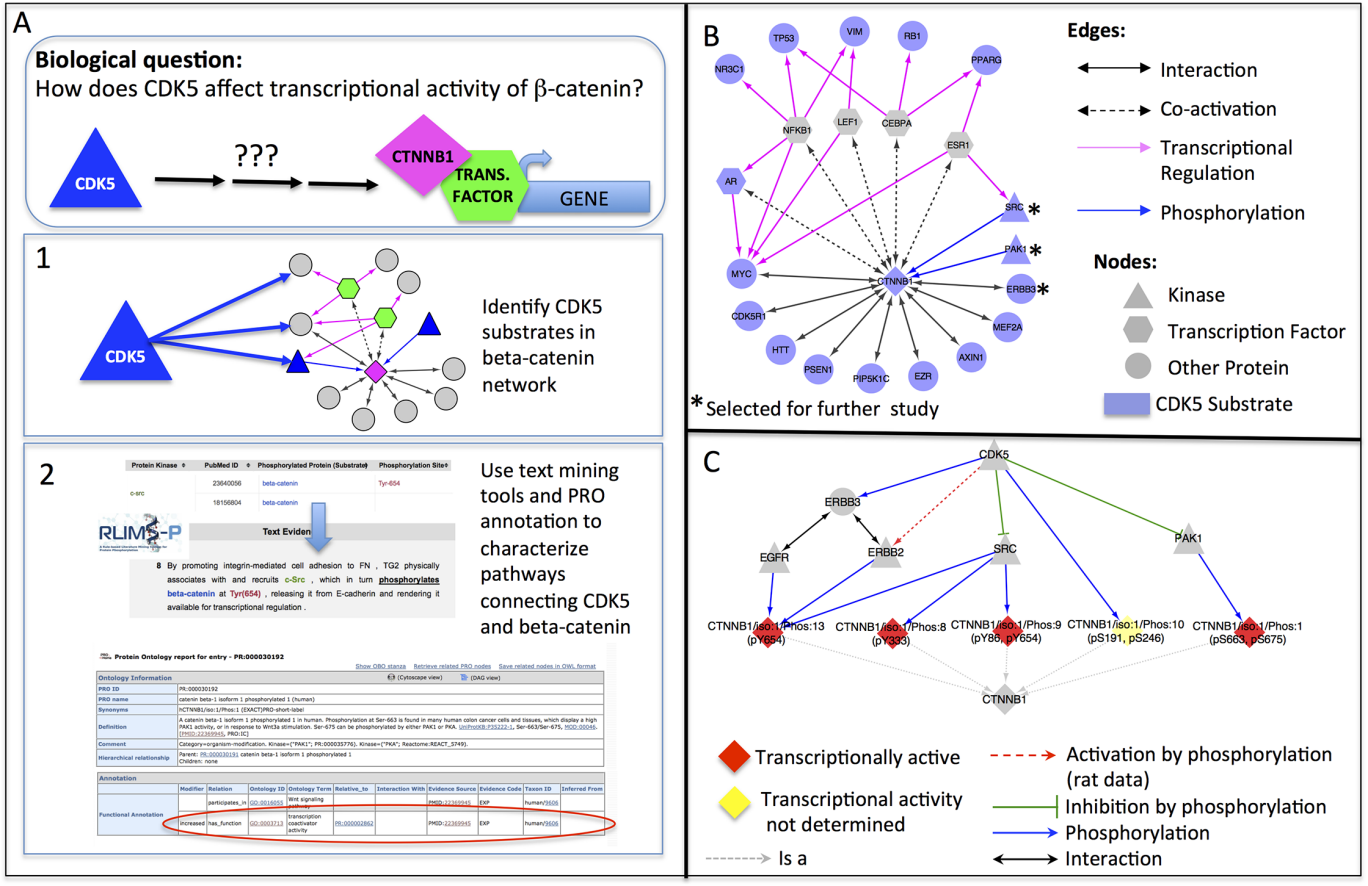
Regulation of beta-catenin activity by kinase signaling. (A) Workflow for exploring effects of CDK5 on beta-catenin transcriptional activity. (B) Sub-network of CDK5 substrates (blue nodes) in the beta-catenin network (Step 1 of workflow) (C) Sub-network of CDK5 substrates selected for further study (Step 2 of workflow). Pathways through which CDK5 kinase activity affects beta-catenin (CTNNB1) phosphorylation state and transcriptional activity are shown.

*CDK5* phosphorylates beta-catenin on Ser-191 and Ser-246 (PR:000037229); however, the effect of this phosphorylation on beta-catenin transcriptional activity has not been reported. While it remains possible that *CDK5* directly regulates beta-catenin transcriptional activity, we looked for evidence that *CDK5* might act indirectly through phosphorylation of another protein in the beta-catenin biological network. The workflow for this analysis is presented in Fig 5A. First, we identified all of the proteins in the beta-catenin network that are reported to be CDK5 substrates in our iPTMnet database. There were 17 such proteins, including two beta-catenin kinases (SRC and PAK1), and ERBB3, a co-receptor for two additional beta-catenin kinases, *EGFR* and *ERBB2*. Moreover, rat *ERBB2* is reported to be a *CDK5* substrate in PhosphoSitePlus [28]. Human and rat beta-catenin are >99% identical and all of the known human beta-catenin phosphorylation sites are conserved; thus, it is plausible that human *ERBB2* can also be phosphorylated by *CDK5*. These findings raise the possibility that *CDK5* can indirectly regulate the phosphorylation of beta-catenin through its influence on other beta-catenin kinases.

We next conducted an in-depth study of the relationships between CDK5, ERBB2, ERBB3, SRC, PAK1, and beta-catenin based on text mining results and PRO functional annotation. As shown in Fig 5B, phosphorylation of beta-catenin by *CDK5* and the kinases it regulates leads to the production of five phosphorylated proteoforms: a Tyr-654 phosphorylated form (PR:000044478, produced by *EGFR*, *ERBB2*, or *SRC*); a Tyr-333 phosphorylated form (PR:000037192, produced by *SRC*); a Tyr-86/Tyr-654 doubly phosphorylated form (PR:000037194, produced by *SRC*); a Ser-191/Ser-246 doubly phosphorylated form (PR:000037229, produced by *CDK5*); and a Ser-663/Ser-675 double phosphorylated form (PR:000030192, produced by *PAK1*). With the exception of the Ser-191/Ser-246 phosphorylated form, PRO annotation indicates that all of these forms are transcriptionally active. We used RLIMS-P to identify sentences in the literature describing phosphorylation of *ERBB2*, *SRC*, and *PAK1* by *CDK5* and manually reviewed the article sections containing the sentences to determine the impact of *CDK5* phosphorylation on substrate activity. Two of the kinases—*PAK1* and *SRC*—are inhibited by *CDK5* and one—*ERBB2*—is activated [28–30]. Thus, *CDK5* may tend to decrease beta-catenin co-activator function through its effects on *PAK1* and *SRC* and increase co-activator function through its effects on *ERBB2*. The net effect of *CDK5* will depend on the levels of these three kinases, their relative affinities for beta-catenin and the cellular context. The fact that *CDK5* promoted beta-catenin transcriptional activity in the siRNA screen suggests that its role in *ERBB2* activation may predominate. Interestingly, *CDK5* has been shown to weaken beta-catenin association with the adherens junction by promoting the phosphorylation of Tyr-654 [31]. Since Tyr-654 is the *ERBB2* phosphorylation site on beta-catenin, this result is consistent with our hypothesis that *CDK5* activates *ERBB2*, which in turn phosphorylates beta-catenin on Tyr-654, leading to a shift of beta-catenin away from the adherens junction and into the nucleus where it can serve as a transcriptional co-activator. Moreover, in cancer cells, *CDK5*, *ERBB2/ERBB3*, and beta-catenin have been linked through another member of the beta-catenin network—the androgen receptor (*AR*). In *ERBB2*-overexpressing breast cancer cells, beta-catenin co-activates *AR* to drive transcription of several tumor-promoting targets, including *ERBB3 {{}}*[32]. As mentioned above, *AR* activation by *CDK5* phosphorylation has been identified as a cancer-driving mechanism in prostate tumors [27]. Taken together with the results of our kinase analysis, these observations suggest that CDK5 phosphorylation of both *ERBB2/ERBB3* and *AR* could drive a feedback loop, in which *ERBB2/ERBB3* promotes beta-catenin transcriptional activity that then contributes to higher expression of *ERBB3*. By combining the kinase and substrate information in multiple phosphorylation databases with phosphorylation-focused mining of the scientific literature, we identified a pathway linking beta-catenin to an upstream kinase, *CDK5*, shown to influence beta-catenin co-activator activity in a kinome-wide siRNA screen.

### Analysis of Cancer-Associated Beta-Catenin Mutations for Cancer Classification

**Fig 6:**
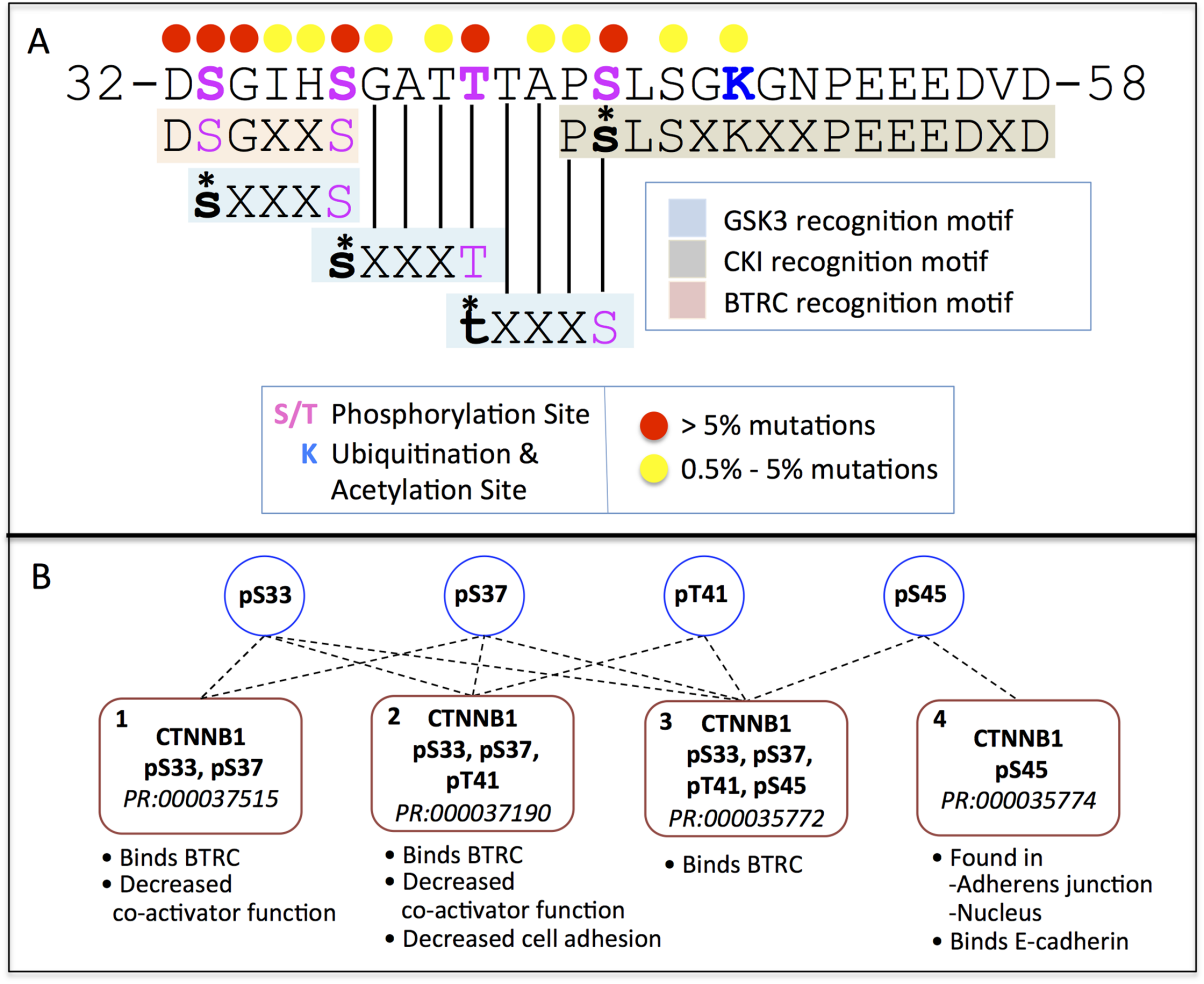
Beta-catenin sequence map of most frequently mutated sites in cancer. PTM sites, PTM enzyme binding sites, and frequencies of cancer-associated mutations at individual sites are indicated. (B) Beta-catenin proteoforms phosphorylated on combinations of the four N-terminal phosphorylation sites Ser-33, Ser-37, Thr-41, and Ser-45 and their functional annotation.

We collected nearly 4,100 cancer-related missense mutations on 137 of 781 residues of beta-catenin and mapped them onto the beta-catenin sequence annotated with sequence features such as PTM sites and PTM enzyme binding motifs. Over 90% of cancer-related mutations occurred in the region encoded by exon 3 of beta-catenin (residues 20 to 60). The top six most frequently mutated sites across all cancer types, accounting for 6–25% of all cancer-associated mutations in beta-catenin (Fig 6A, red dots) include the CKI and *GSK3B* phosphorylation sites (Ser-45, Thr-41, Ser-37, and Ser-33) and two highly conserved residues in the *BTRC* ubiquitin ligase recognition motif (Asp-32 and Gly-34). Several proteoforms phosphorylated on different combinations of the four highly mutated phosphorylation sites have been described in the literature (Fig 6B). While three of the four proteoforms (Fig 6B, forms 1, 2, and 3) bind *BTRC*, rendering them unstable, the fourth proteoform, phosphorylated on Ser-45 only (Fig 6B, form 4, PR:000035774), is found at the adherens junction in association with E-cadherin and in the nucleus, suggesting it may play in active role in adhesion and/or transcriptional regulation [33].

**Fig 7:**
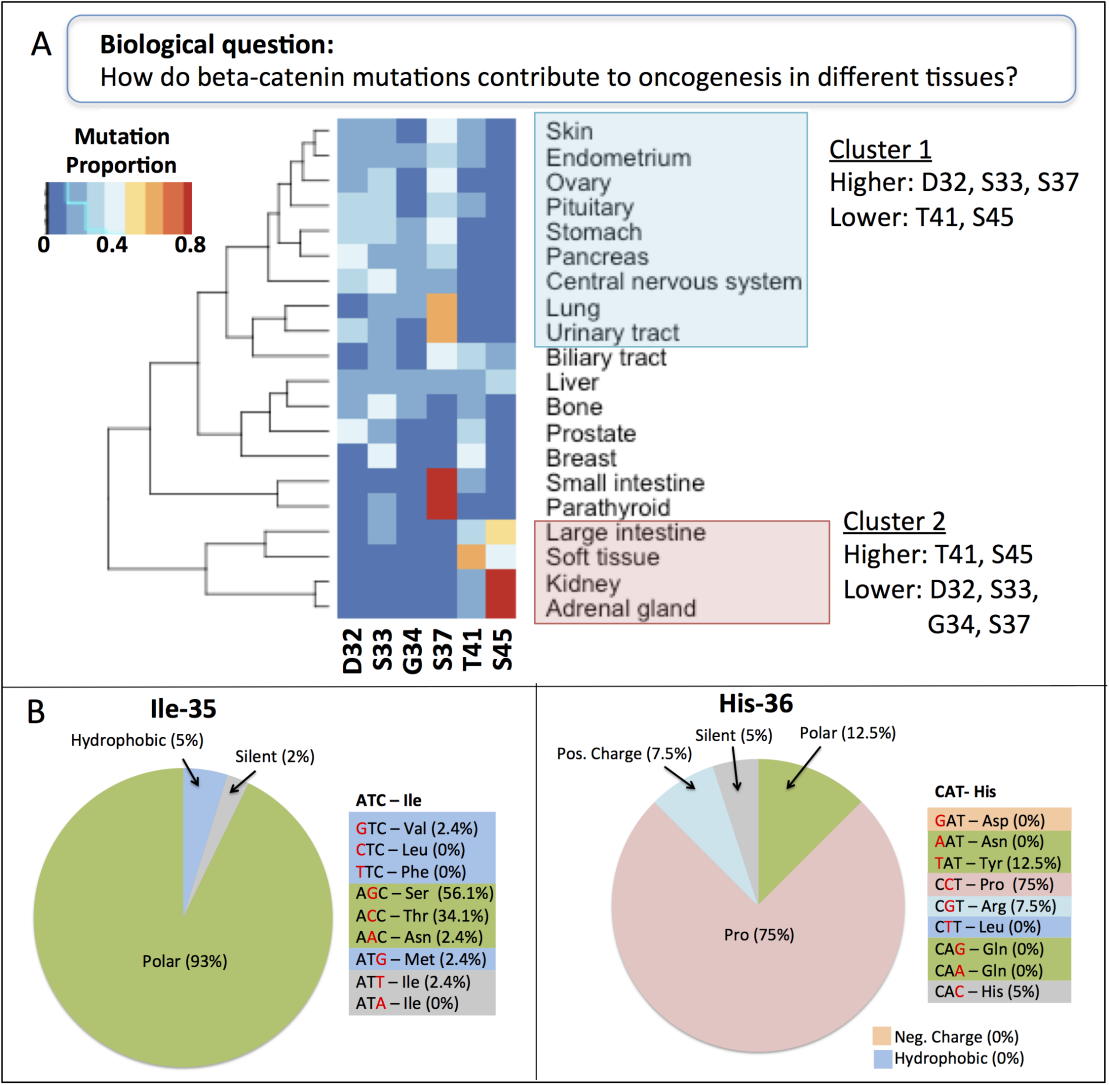
Mutation patterns of beta-catenin across cancer types. (A) Hierarchical clustering of cancer tissues based on their pattern of mutations in the six most frequently mutated beta-catenin residues in cancer: Asp-32, Ser-33, Gly-34, Ser-37, Thr-41, and Ser-45. (Band C) Frequencies of the various possible amino acid substitutions at beta-catenin residues Ile-35 (B) and His-36 (C) observed in cancer samples. Amino acids are color coded and grouped in the pie charts according to the chemical nature of their side chains.

To investigate the pattern of mutation frequencies across individual cancer types, we performed hierarchical clustering analysis for 20 different tissues based on their distribution of cancer-associated mutations across the six most frequently mutated sites in cancer overall (Asp-32, Ser-33, Gly-34, Ser-37, Thr-41, and Ser-45). Since the overall frequency of mutations of these sites is similar we assume similar frequency of mutation in the sampled tissues. However, we found two clusters with highly distinct mutation profiles emerged from this analysis (Fig 7A). Cluster 1 consists of nine tissues with mutations predominantly in Asp-32, Ser-33, and Ser-37 and relatively few mutations in Thr-41 and Ser-45 (Fig 7A, blue box). In contrast, Cluster 2 consists of four tissues with mutations in Thr-41 and especially Ser-45 with few mutations in the *BTRC* binding region (Asp-32, Ser-33, Gly-34, and Ser-37; Fig 7A, pink box). The same general pattern was also observed in a clustering analysis considering just the four phosphorylation sites [34]. One possibility is that these clusters arise because the mutagenic environment in different tissues varies, leading to different patterns of DNA base changes as has been observed for p53 [35]. However, we found that in all tissues a variety of base changes were responsible for the observed mutations in the six beta-catenin N-terminal sites; in most cases, each amino acid substitution was caused by multiple DNA base changes at multiple positions within the codon (S2 Table). For example, Ser-45 mutations in adrenal cancers are due to T to C mutations at the first position of the codon as well as C to T and C to A mutations at the second position. These results suggest that biases in the nature and location of DNA mutations are unlikely to explain the amino acid mutation patterns.

Another possibility is that the mechanisms of beta-catenin induced oncogenesis differ between the tissues in the two clusters. Oncogenesis in Cluster 1 tissues, which have high proportion of mutations in the *BTRC* binding sites, may be driven by loss of *BTRC* binding and beta-catenin stabilization as the canonical model proposes. However, these tissues have a low mutation frequency at the priming phosphorylation sites, Thr-41 and Ser-45. Assuming that Thr-41 and Ser-45 mutations occur in these tissues at the same rate as in the overall set, the relative absence of these mutations in cancer samples suggests that these mutations are not strong cancer drivers in these tissues. Thus, phosphorylation of Ser-33 and Ser-37 may be independent of upstream priming phosphorylation events. A beta-catenin proteoform phosphorylated on Ser-33 and Ser-37 capable of binding *BTRC* has been reported; this form is phosphorylated by *HIPK2* kinase, which does not require upstream priming (Fig 6B, form 1, PR:000037515). In the Cluster 2 tissues, loss of the *BTRC* binding site does not appear to play a major role; instead, these tissues are sensitive to the loss of Ser-45. The Ser-45 phosphorylated beta-catenin proteoform has been observed in the nucleus and at the adherens junction (Fig 6B, form 4, PR:000035774), suggesting that cancer in these tissue may arise from qualitative changes in the activity of the nuclear and cell membrane pools of beta-catenin. The remaining tissues had various distributions of mutations across the six sites. Small intestine and parathyroid cancers had particularly high rates of mutations in Ser-37, suggesting the possibility of a third cluster. Our analysis highlights the need for experimental studies at both the DNA and protein level to understand the differences in cancer-associated mutation patterns in the two tissue clusters we observed.

Eight additional residues in the beta-catenin exon 3 region each account for at least 0.5% of the total cancer-associated mutations. Two of these residues—Ile-35 and His-36—lie at less conserved positions of both the *BTRC* and *GSK3B* recognition motifs. The distribution of amino acid changes resulting from single base changes in the Ile-35 and His-36 codons in cancer cells is shown in Fig 7B and 7C. The four single base changes in the Ile-35 codon that result in a switch from hydrophobic Ile to a different hydrophobic amino acid (Val, Leu, Phe, Met) rarely appear in cancer cells. In contrast the two mutations that cause a switch to a polar amino acid (Ser or Thr) are very common. In agreement with these observations, structural studies of the interface between beta-catenin and *BTRC* demonstrate that the side chain of Ile-35 makes several van der Waals contacts with *BTRC*, so its hydrophobic nature is likely to be important [36]. At position 36, the one base change that introduces a Pro accounts for three-quarters of the cancer-associated mutations. In the beta-catenin/*BTRC* structure, the side chain of His-36 points away from *BTRC* and does not interact, suggesting that most substitutions should be tolerated here. However, the amino group of His-36 does participate in *BTRC* binding; proline, with its secondary amine group, is the one amino acid that would be least likely to successfully make these contacts. The *GSK3B* recognition motif indicates that any residue is tolerable at both of these positions and inspection of the residues surrounding the phosphorylation site of a large number of *GSK3B* substrates in phosphorylation databases confirms that indeed a wide range of residues can occupy these positions (data not shown). Taken together, these results suggest that the high frequency of Ile-35 and His-36 mutations in cancers is likely to reflect impairment of *BTRC* binding to beta-catenin.

## Discussion

In this paper, we presented a beta-catenin knowledge map generated by capturing information about PTM, PPI, transcriptional co-activation and transcriptional targets, as well as beta-catenin disease-associated mutations, sequence features, and functional information. Publicly available text-mining and data-mining tools enabled us to integrate and analyze this wide variety of knowledge. Our approach helped us to gain insight into the roles of beta-catenin in healthy and cancerous cells.

Such knowledge integration can support scientific research and lead to novel discoveries, as illustrated by our beta-catenin study. First, it is a mechanism for organizing information about a topic that is otherwise scattered, providing a comprehensive, integrated picture of what is known about a protein. For example, it is possible to easily identify proteins that are connected to the protein of interest by more than one type of relation. In our study, we analyzed proteins that were both (i) beta-catenin interacting proteins and (ii) potential beta-catenin transcriptional targets (i.e., targets of a transcription factor co-activated by beta-catenin). These are proteins whose expression may be regulated by beta-catenin that, in turn, modulate beta-catenin activity in some way. By looking at the effects that these proteins have on beta-catenin transcription function, we found several candidates that may participate in positive or negative transcriptional feedback loops. Because transcription factor-target relations and PPIs are most often reported in separate articles or stored in separate databases, proteins that share both relationships are not readily apparent without knowledge integration.

Additionally, we can generate hypotheses to explain experimental findings, pointing the way to future experiments. For example, the development of high-throughput siRNA-based knock-down screening methodologies has produced data on gene product-phenotype connections in mammalian cells on a scale that was previously possible only in simpler model organisms. However, as in classic mutational screening approaches, extensive follow-up studies are often necessary to understand the molecular mechanism by which the knock-down of a gene product leads to the observed cellular phenotype. A biological network can be used to explore knowledge gaps and guide these follow-up studies by identifying pathways that connect the perturbed gene product with molecules that could be mediating the phenotype. Using our beta-catenin data, we proposed a chain of events linking *CDK5*, the *ERBB2/ERBB3* receptor tyrosine kinase, and beta-catenin that could explain the inhibitory effect of *CDK5* siRNA knock-down on beta-catenin co-activator activity. The individual pieces were already known: *CDK5* was known to positively regulate *ERBB2/ERBB3* by phosphorylation; *ERBB2/ERBB3* were known to phosphorylate beta-catenin on Tyr-654; and Tyr-654 phosphorylation was known to stimulate beta-catenin co-activator function at the expense of its cell adhesion function. However, viewing these pieces side-by-side made it possible to see how they could fit together into a single pathway.

Finally, examining beta-catenin mutations across cancer types and at particular residue positions (e.g. Ile-35 and His-36) revealed interesting patterns. By overlaying this information with beta-catenin sequence features and PTM proteoform-specific functional information, we were able to propose explanations for how the different mutation patterns could contribute to dysfunction of beta-catenin and disease. Ontologies such as PRO, which supports the definition of PTM proteoforms and GO, which provides a structured format for functional annotation, were particularly helpful for the interpretation of mutations in the N-terminal phosphorylation sites of beta-catenin because we could easily see which proteoforms, and which associated beta-catenin functions were affected in each cluster of cancers.

Knowledge integration poses several challenges. One concern is quality control of the integrated data. The quality of the constructed networks and the scientific conclusions drawn from then depend heavily on the quality of the molecular interactions that comprise them. Many of the resources used to build our knowledge network, such as STRING and the Transcriptional Regulatory Element Database (TRED), employ a scoring system for assigning confidence to the relations they contain. For our beta-catenin network, we set moderate cutoff scores (e.g., STRING scores > 0.6) to strike a balance that limited inclusion of inaccurate relationships while still creating a comprehensive view of the cellular roles of beta-catenin. In general, confidence cut-offs can be set more or less stringently depending on user needs. We also address the issue of data quality by using manually curated databases. Currently, we manually verify all of our text-mining results; however, we are working toward further automation by developing systems to assign confidence to relations extracted by text-mining. These systems will take into account the article section where the relation is found (e.g., a statement in the Abstract or Conclusion would be assigned higher confidence than a statement in the Introduction or Results, which could be describing a hypothesis or experimental set-up) as well as the language used by the authors in stating the relations (e.g., whether the statements contains words indicating hedging or speculation).

Another challenge is that most resources express molecular interactions in terms of gene-level entities using the gene symbol or UniProtKB accession number to identify the participants, thereby obscuring any proteoform-specific effects. PRO, which provides a framework for representing the multiple proteoforms that arise from a single gene, can play a key role in addressing this issue. In this study, we have associated PTM proteoforms of beta-catenin defined in PRO with their modifying enzymes and with proteoform-specific functional information. Going forward, usage of text-mining tools will be valuable for retrieving in depth information about proteoform-specific molecular relations. For example, we have developed the Extracting Functional Impact of Phosphorylation (eFIP) text-mining tool to detect effects of phosphorylation on PPI; extension to other impacts, such as changes in enzymatic activity are planned [37].

The time and effort required to extract information from the disparate resources used in this study highlighted the desirability of creating an integrated query interface that enables browsing, searching, and visualization of a wide range of gene, protein, disease, and drug information from a single web portal with knowledge derived from multiple sources. Toward that end, we are continuing to develop our iPTMnet database and web portal (http://proteininformationresource.org/iPTMnet/). Using information text-mined from the literature as well as information in curated PTM resources, iPTMnet links enzyme-substrate relationships, ontologies, and functional impacts, such as PPIs, and provides a visualization of these PTM relationships. Finally, the ability to conduct relation-centric (as opposed to entity-centric) searches allows users to explore the relations that connect their genes/proteins of interest. By linking together multiple relation-centric searches, genes/proteins connected by complex relation types can be identified. Most other integrated resources such as UniProtKB [38] and GeneCards [39] represent information in an entity-centric manner. A relation-centric approach that emphasizes the relationship between genes/proteins would offer users a unique perspective on information integrated from dispersed sources.

The number of curated and publicly available databases has been growing rapidly. These databases provide up-to-date knowledge about a variety of protein relations and properties. Using our approach, scientists can assemble a comprehensive picture of their protein of interest in health and disease. Analysis of this data can reveal missing links in the current knowledge and generate hypothesis to direct future experimental work.

## Methods

### Knowledge Integration and Visualization

To construct the beta-catenin knowledge map, text mining results were integrated with information from biomedical ontologies and curated databases, according to the general outlined in Fig 1. Details of the various information sources used are given below. All data were imported into a local relational database. The molecular relations and cancer-associated mutations used in this study can be found in S1 Table and S3 Table, respectively.

Information about phosphorylation of human beta-catenin was retrieved from the literature by querying the text mining tool, RLIMS-P [9] with the terms "beta-catenin" and "human". Out of >10,000 articles retrieved by PubMed with these keywords, RLIMS-P identified approximately 1,600 as containing potential phosphorylation information. For those results where the phosphorylated substrate was beta-catenin, we consulted the cited article to validate the information provided by RLIMS-P and to collect further information about the function of the phosphorylated forms. We mapped the forms to existing PRO terms and for those forms which did not already exist in PRO, we created new terms and annotated these with functional information using GO terms and kinase information as described in [1,40]. All terms and annotations are disseminated in the PRO website (http://proconsortium.org/) and in files downloadable as part of PRO release 45 (April 2015).

Additional information about beta-catenin PTM sites, PTM enzymes, and PTM enzyme binding motifs was obtained from dbPTM (http://dbptm.mbc.nctu.edu.tw/) [41] and our iPTMnet database (http://proteininformationresource.org/iPTMnet/), which integrates information from PhosphoSitePlus (http://www.phosphosite.org/) [2], Phospho.ELM (http://phospho.elm.eu.org/) [11], and Human Protein Reference Database (http://www.hprd.org/) [42], as well as several plant and yeast PTM resources. Only the experimental (as opposed to predicted) results were taken from these databases to reduce the chance of false positives.

To identify the transcriptional factors co-activated by beta-catenin, we used Dragon Database of Transcription Co-factors and Transcription Factor Interacting Proteins (TcoF; http://cbrc.kaust.edu.sa/tcof/) [43]. Of nineteen transcription factors binding to beta-catenin, we selected *AR*, *ESR1*, *CEBPA*, *NFKB1*, and two TCF/LEF family members (*LEF1* and *TCF7L2*), due to the evidence that they are co-activated by beta-catenin. Target genes of for five of these transcription factors (*AR*, *ESR1*, *CEBPA*, *NFKB1*, and *LEF1*) were obtained from TRED; http://rulai.cshl.edu/cgi-bin/TRED/; parameters: Binding Quality—1:Known, Promoter Quality: 1: Known-curated, known, 2: Refseq-predicted and 3: Refseq); no target genes were available for *TCFL2* in TRED. We downloaded human beta-catenin interacting proteins from the STRING database v.9.05 (http://string-db.org/; parameters: data sources = Experiments and Databases; confidence > 0.6) [18]. Information on missense mutations in beta-catenin observed in cancers of various types was gathered from Catalogue of Somatic Mutations (COSMIC; http://cancer.sanger.ac.uk; [44].

Network views were created using Cytoscape v3.1.1 [45].

### Functional Annotation Clustering

Functional annotation clustering of GO Biological Process terms was performed using the DAVID web interface (http://david.abcc.ncifcrf.gov/) [12] with default parameters. Enriched terms with Benjamini-Hochberg score < 0.05 (up to a maximum of ten terms per cluster) from the ten highest scoring clusters are displayed in the treemap. For the biological network as a whole (Fig 3A), the enrichment scores for the top ten clusters ranged from 17.8 to 52.0; for the target-interactors (Fig 3B) scores ranged from 3.2 to 9.0. Names for the clusters were chosen manually based on the predominant process among the terms in the cluster. The treemap was created using the R (version 3.0.3; http://www.r-project.org/) treemap function. In the treemap, supercategories (colored blocks) correspond to functional clusters; the sizes of the individual term boxes are determined by the p-values for the enrichment.

### Analysis of Cancer-Associated Mutations

To determine the proportion of cancer-associated mutations at each site in beta-catenin (Fig 6), we divided the number of missense mutations at each site by the total number of beta-catenin missense mutations at all sites. For the cancer clustering analysis (Fig 7A), we used mutation data from COSMIC database (time range between June-November 2013) for all cancer tissues that had at least 10 samples with mutations in the six sites of interest (Asp-32, Ser-33, Gly-34, Ser-37, Thr-41, and Ser-45). For each tissue, we calculated the proportion of missense mutations at each site relative to the total number of missense mutations at all six sites. The heatmap was constructed using the heatmap.2 function of R (version 3.0.3; http://www.r-project.org/) with default parameters. For the analysis of Ile-35 and His-36 substitutions (Fig 7B and 7C), we considered all possible single base change at any of the three positions in the codon. Using missense mutation data from COSMIC, we calculated the proportion of each base change relative to the total number of missense mutations affecting the codon.

## Supporting Information

S1 Table Molecular Relations in the Beta-Catenin Network. (XLSX)

S2 Table Frequencies of Cancer-Associated DNA Base Mutations at Selected Sites in Beta-Catenin. (XLSX)

S3 Table Cancer-Associated Mutations in Beta-Catenin. (XLSX)

## Abbreviations

COSMIC: Catalog of Somatic Mutations
eFIP: Extracting Functional Impact of Phosphorylation
GO: Gene Ontology
PPI: Protein-Protein Interaction
PRO: Protein Ontology
PTM: Post-Translational Modification
RLIMS-P: Rule-based Literature Mining System for Protein Phosphorylation
siRNA: small interfering RNA
STRING: Search Tool for the Retrieval of Interacting Genes/Proteins
TCF/LEF: T-Cell Factor/Lymphoid Enhancing Factor
TcoF: Dragon Database of Transcription Co-factors and Transcription Factor Interacting Proteins
TRED: Transcriptional Regulatory Element Database
